# Application of Metabolomics in Alzheimer’s Disease

**DOI:** 10.3389/fneur.2017.00719

**Published:** 2018-01-12

**Authors:** Jordan Maximillian Wilkins, Eugenia Trushina

**Affiliations:** ^1^Mitochondrial Neurobiology and Therapeutics Laboratory, Department of Neurology, Mayo Clinic, Rochester, MN, United States; ^2^Department of Molecular Pharmacology and Experimental Therapeutics, Mayo Clinic, Rochester, MN, United States

**Keywords:** Alzheimer’s disease, metabolomics, lipidomics, biomarkers, animal models of Alzheimer’s disease

## Abstract

Progress toward the development of efficacious therapies for Alzheimer’s disease (AD) is halted by a lack of understanding early underlying pathological mechanisms. Systems biology encompasses several techniques including genomics, epigenomics, transcriptomics, proteomics, and metabolomics. Metabolomics is the newest omics platform that offers great potential for the diagnosis and prognosis of neurodegenerative diseases as an individual’s metabolome reflects alterations in genetic, transcript, and protein profiles and influences from the environment. Advancements in the field of metabolomics have demonstrated the complexity of dynamic changes associated with AD progression underscoring challenges with the development of efficacious therapeutic interventions. Defining systems-level alterations in AD could provide insights into disease mechanisms, reveal sex-specific changes, advance the development of biomarker panels, and aid in monitoring therapeutic efficacy, which should advance individualized medicine. Since metabolic pathways are largely conserved between species, metabolomics could improve the translation of preclinical research conducted in animal models of AD into humans. A summary of recent developments in the application of metabolomics to advance the AD field is provided below.

## Introduction

Alzheimer’s disease (AD) is the leading cause of dementia resulting in memory loss, difficulty with thinking, and behavioral changes (1). Patients with early-onset familial AD (FAD) carry mutations in genes coding for amyloid precursor protein (APP; chromosome 21), presenilin-1 (PS1; chromosome 14), and presenilin-2 (PS2; chromosome 1) (2). Each of these mutations results in an increased production of amyloid-β (Aβ) peptides (3–6). However, the majority of AD cases are sporadic (>95% prevalence) without a specific genetic link. Individuals carrying the ε4 allele of apolipoprotein E (APOE), a lipid transport protein, have increased risk for developing AD at a younger age (7). Approximately 25% of AD patients in the United States carry one or more copy of the APOE ε4 allele (8). The disease is progressive with age being the greatest risk factor (9). According to the Alzheimer’s Association, nearly two-thirds of Americans with AD are females (10). Women who are positive for the APOE ε4 allele are at greater risk of developing AD compared to men with the same variant (11). Additionally, female APOE ε4 carriers develop more severe behavioral changes compared to men (12). However, the molecular mechanisms behind these differences remain to be elucidated. To date, strategies approved for AD treatment provide only symptomatic relief for some individuals. Disappointingly, recent clinical trials that focused on the prevention of Aβ production have consistently failed (13). Lack of success may be related to the enrollment of participants into clinical trials at a point when it may be too late to reverse or stop disease progression. Furthermore, several Aβ-independent mechanisms including impaired calcium and lipid homeostasis, mitochondrial dysfunction, altered cell signaling, synaptic transmission, oxidative stress, and inflammation have been shown to contribute to AD pathogenesis (13). Therefore, targeting Aβ alone may not be sufficient to achieve therapeutic efficacy, especially during late stages of the disease (14). Indeed, it became increasingly recognized that cellular decline in AD patients begins up to 20 years prior to the manifestation of clinical symptoms. As AD progresses, multiple pathways are synergistically affected activating a vicious cycle that ultimately devastates neuronal formation of synapses resulting in declined cognitive function (15). Interestingly, metabolic decline is one of the earliest symptoms detected using fluorodeoxyglucose positron emission tomography (FDG-PET) in patients with mild cognitive impairment (MCI), an early stage of AD (16). This suggests that metabolism could play an essential role in early AD mechanisms. Depending on the stage of AD and individual traits (e.g., age, sex, race, etc.), treatment options may vary and most likely will require combinatorial therapy (17, 18). Thus, novel technologies for the unbiased detection of changes associated with early disease mechanisms could be instrumental in the development of biomarkers for preclinical and clinical diagnosis, prognosis, and monitoring the outcome of treatment.

Metabolomics is the newest systems biology approach where multiple platforms are utilized to measure levels of small molecule metabolites in biological samples (19). Metabolic signatures are unique to an individual wherein perturbations in metabolite levels may inform on the disease state and underlying mechanisms of the disorder. The strength of metabolomics is in its ability to identify dynamic, qualitative, and quantitative changes in a large number of metabolites (in the thousands) representing alterations in multiple functional networks. Indeed, Oliver et al. first used the term metabolome to emphasize the importance of measuring changes in metabolite concentrations as a result of altered gene expression (20). Initially utilized as a high-throughput analytical tool primarily in studies related to toxicology, metabolomics has become increasingly used to research metabolic perturbations in multiple human diseases including AD. The availability of biorepositories, such as Coriell Cell Repository and Alzheimer’s Disease Neuroimaging Initiative (ADNI), which contain biofluids and tissue samples from control (CN) and AD patients provide an outstanding opportunity to advance the understanding of sex- and disease-specific metabolic signatures and mechanisms. As blood is a readily available biofluid for recurrent measures, longitudinal studies using metabolomics could significantly enhance the precision of individualized medicine. Moreover, since metabolic pathways are largely conserved between species, application of metabolomics could provide a strong tool to translate experimental findings in preclinical mouse models to humans. Below we review recent applications of metabolomics to develop disease biomarkers, conduct preclinical drug discovery, and advance our knowledge of the etiology and pathogenesis of AD.

## Metabolic Decline in AD

**Figure 1:**
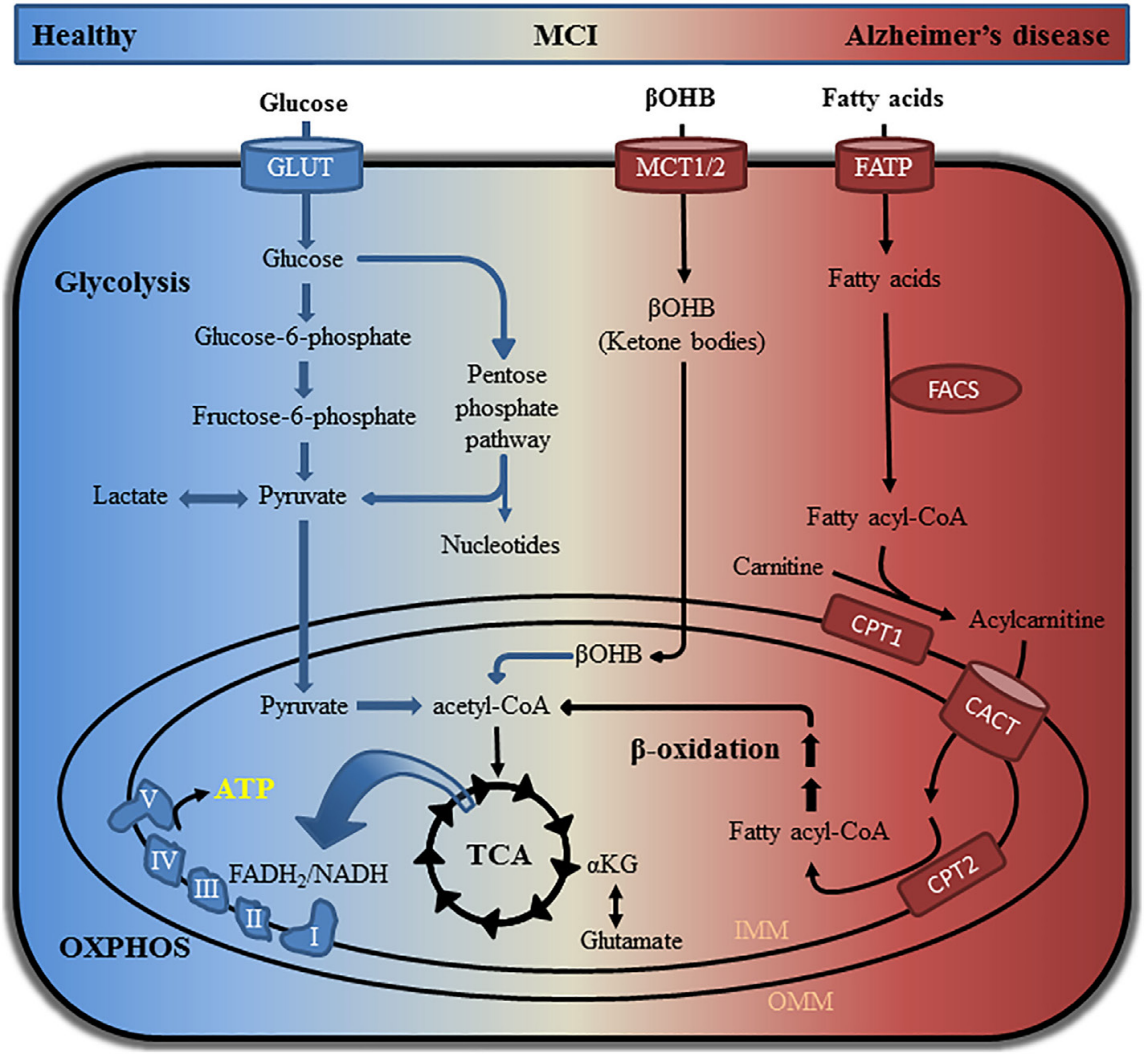
Pathways involved in glucose, ketone body, and lipid metabolism. Glucose can be catabolized *via* glycolysis or the pentose phosphate pathway to produce intermediate metabolites that promote cell growth and function. Oxidation of glucose generates pyruvate, which is shuttled into mitochondria where it is converted to acetyl-CoA. Utilization of acetyl-CoA in the TCA cycle generates several intermediates that can be used for nucleotide, lipid, and amino acid synthesis. Electrons from the reducing equivalents NADH and FADH_2_ are used for oxidative phosphorylation (OXPHOS) to generate ATP. Healthy neurons are highly glycolytic catabolizing glucose *via* glycolysis and the TCA cycle in order to produce ATP through OXPHOS. Metabolic instability and decreased glucose utilization in AD patients can be detected by metabolomics approaches and fluorodeoxyglucose positron emission tomography. Impaired glycolytic processes in the brain can cause a shift toward the use of alternative fuel sources including ketone bodies and fatty acids. Processing of ketone bodies and fatty acids can produce acetyl-CoA for use in the TCA cycle and OXPHOS. αKG, alpha-ketoglutaric acid; βOHB, β-hydroxybutyric acid; CACT, carnitine acylcarnitine translocase; CPT1/CPT2, carnitine palmitoyltransferase 1/2; FACS, fatty acyl-CoA synthetase; FADH2, flavin adenine dinucleotide + hydrogen (H); FATP, fatty acid transport protein; GLUT, glucose transporter; IMM, inner mitochondrial membrane; MCT1/MCT2, monocarboxylate transporter 1/2; NADH, nicotinamide adenine dinucleotide (NAD) + hydrogen (H); OMM, outer mitochondrial membrane; TCA, tricarboxylic acid.

In patients with MCI, a prodromal stage of AD (21, 22), early abnormalities are associated with reduced glucose utilization detected using FDG-PET (23, 24). This brain hypometabolism occurs ~20 years prior to the manifestation of clinical symptoms suggesting that metabolic dysfunction is a contributing factor for AD development (6–9). The brain is highly dependent on glucose consuming approximately 20% of total glucose-derived energy while accounting for about 2% of body weight (25). When glycolytic functions in the brain are perturbed, compensatory mechanisms switch to alternative fuel sources to maintain energy homeostasis (Figure 1) (26). Indeed, white matter degeneration in the brain tissue of aged wild-type mice is linked to the catabolism of lipids in order to compensate for reduced glucose utilization, which could be similar to that observed in the aging female brain (27). Supplementation with ketone bodies in AD transgenic mice and patients suggest that a ketogenic diet may improve cognition (27–29). Studies, including our own, have shown unique metabolic signatures associated with altered energy homeostasis in plasma and cerebrospinal fluid (CSF) of patients with MCI, which became more pronounced in patients with AD (30–32). Metabolic networks perturbed early in MCI individuals included lysine metabolism, tricarboxylic acid (TCA) cycle, lipid metabolism, and mitochondrial ketone bodies when compared to healthy individuals (30). In AD patients, metabolic alterations in multiple networks including neurotransmission and inflammation were detected in both CSF and plasma; however, the most pronounced changes in energetic pathways remained (30). Similar findings were also observed in multiple mouse models of AD where changes in metabolic pathways related to energetic stress in female mice were greater compared to males (33). By identifying longitudinal changes in the metabolic networks of CN, MCI, and AD patients, it is possible to establish panels of metabolic biomarkers and gain valuable mechanistic insight into disease mechanisms. With the recent failure of clinical trials aimed to modulate Aβ production, the attention in preclinical drug discovery and academic research has shifted toward the identification of new therapeutic targets and early mechanisms of AD including altered brain energetics and mitochondrial dysfunction (34). As metabolomics allows monitoring changes in multiple connected networks essential for understanding complex metabolic alterations, its application in AD research is gaining momentum.

## Analytical Platforms

Metabolomics encompasses several techniques including untargeted metabolomics, targeted metabolomics, lipidomics, and fluxomics (35–37). Untargeted metabolomics measures hundreds of metabolites in order to identify metabolic signatures related to a particular disease state or phenotype. This approach provides relative changes in metabolites and is useful for discovery projects where affected metabolic pathways are unknown. Targeted metabolomics provides quantitative measurements of a defined set of metabolites in a pathway of interest (e.g., glycolysis or TCA cycle). Lipidomics estimates changes in lipid profiles and requires specialized protocols for the detection and analysis of water-insoluble metabolites. Fluxomics incorporates stable isotope tracers to provide a dynamic, as opposed to static, assessment of metabolic changes and is done in cells or *in vivo*. Most often, heavy carbon (^13^C) precursors are introduced into a system, which can be traced through a metabolic pathway by measuring different mass isotopomers providing insight into the flux (rate) through a specific network. Each of these applications can be done in a variety of samples including cells, tissue, and biofluids.

Metabolites are small molecules (<1,500 Da) implicated in most biological functions (38). The human metabolome is estimated to contain approximately 150,000 or more metabolites (39). Currently, the Human Metabolome Database contains over 100,000 metabolite entries (40). While a large fraction of human metabolites are unidentified, significant efforts are dedicated toward their discovery and identification. Due to the complex nature of the human metabolome and the diversity of sample composition, several analytical platforms have been adopted for proper detection of each group of metabolites. Mass spectrometry (MS) and nuclear magnetic resonance (NMR) spectroscopy are two analytical platforms regularly used for detection, quantification, and characterization of metabolites. A comparison of NMR and MS is outlined in Table 1.

**Table 1:** Analytical platforms utilized for metabolomics research.

	Nuclear magnetic resonance	Mass spectrometry
Platform cost	High	Moderate

Analysis	Untargeted analysis Reproducibility is high	Targeted analysis Untargeted analysis Reproducibility is moderate

Sample preparation	Minimal preparation Can be directly applied to biofluids and intact tissues Sample recovery is possible	Moderate preparation Metabolite extraction is usually required GC-MS is volatile and typically requires derivatization LC-MS can form adducts

Sensitivity	Low detection range (micromolar) Requires protonated compounds Detects most organic molecules	High detection range (femtomolar) Detects most organic molecules Detects some inorganic molecules

Measurements	Detects all metabolites in a single measurement within detectable range Spectral analysis is demanding	Requires multiple techniques for a comprehensive analysis Has broader range of metabolite detection

Nuclear magnetic resonance spectroscopy is a quantitative non-destructive technique that provides detailed information on molecular structure. Several advantages of NMR include minimal sample preparation, high-throughput capability, low cost per sample, and excellent data acquisition and reproducibility (41). However, NMR has relatively low sensitivity and limited detection of low molecular weight molecules (42). The use of high-field magnets, cryogenically cooled probes, and microcoil detectors can increase sensitivity (43, 44) but usually leads to longer times of analysis.

Mass spectrometry-based metabolomics offers higher sensitivity (femtomole levels) and the capability of detecting a broader range of metabolites. Biological samples are typically separated prior to MS analysis using various chromatography techniques including gas chromatography (GC) (45), liquid chromatography (LC) (46, 47), ultraperformance LC (UPLC) (48), high pressure LC (47), capillary electrophoresis/electrospray ionization (49), Fourier transform (FT) infrared spectroscopy (50), and FT ion cyclotron resonance (51). Chromatography coupled to MS offers a multidimensional analysis providing data related to chemical structure, mass, isotope distribution, and detection of unidentified molecules (52). For these reasons, LC- and GC-MS are often the preferred platforms. However, LC- and GC-MS require elaborate sample preparation, which can reduce metabolite recovery (53). Additionally, separation of molecules increases the time of analysis involving multiple runs for a large batch of samples leading to inter-batch variation, which is a common issue for MS-based metabolomics. Furthermore, while the selectivity of LC- and GC-MS (non-volatile and volatile, respectively) improves sensitivity, parallel application of the techniques is often required for a comprehensive analysis of the metabolome.

## Application of Metabolomics in Human Samples

One of the most promising applications of metabolomics is the development of biomarker panels to detect alterations in multiple interconnected networks. Metabolomics-based biomarkers could provide comprehensive information compared to a single metabolite approach (54). To date, the definitive diagnosis of AD can only be done by postmortem examination of brain tissue for the presence of Aβ plaques and neurofibrillary tangles (NFTs) composed of hyperphosphorylated microtubule-associated protein tau (MAPT; p-tau) (55). Provisional diagnosis of AD relies on the results of neuropsychological tests and appearance of typical symptoms of the disease (56). The use of biomarkers such as levels of Aβ, p-tau, and t-tau (total-tau) in the CSF together with brain imaging using positron emission tomography with Pittsburgh compound B (PiB-PET) or magnetic resonance imaging increases the accuracy of diagnosis and helps to discriminate between different types of dementia. However, these tests are expensive. Furthermore, collection of CSF is associated with some health risk and may not be suitable for recurring testing. Therefore, the development of a simple, safe, and accurate test in readily available biological fluids, such as blood, is of great importance. In our earlier review, we described metabolomics studies performed prior to 2013 (57). Here, we summarize the most recent work (Table 2).

**Table 2:** Application of metabolomics in samples from MCI and AD patients.

Analytical platform	Samples	Findings	Reference
UPLC-HILIC-MS and ionKey/MS	Frontal cortex from 21 AD and 19 CN	Glycerophospholipid predominately altered in AD cortex ↑ NAA in AD cortex Mitochondrial dysfunction and aspartate metabolism correlated with dementia and AD pathology	(58)

HILIC LC-MS and GC-MS	Cerebellum (little AD pathology), middle frontal gyrus (increased AD pathology), inferior temporal gyrus (increased tau pathology) from 14 AD, 14 CN, and 15 asymptomatic (display AD pathology without dementia)	Global brain UFA perturbations as well as region-specific alterations in AD patients Within middle frontal gyrus ↓ Linoleic acid, linolenic acid, and arachidonic acid (CN > ASYMAD > AD) and ↑ docosahexanoic acid (AD > ASYMAD > CN) may serve as regional threshold markers associated with Aβ plaques, tau tangles, and cognitive decline	(59)

Biocrates Absolute IDQ p180 Kit measured using FIA-MS/MS and HPLC-MS/MS	CSF from 50 AD-like (↓ Aβ42, ↑ t-tau and p-tau) and 50 CN	Two SM, five glycerophospholipids, and one AC were significantly altered in CSF with pathological Aβ and tau levels ↑ SM (d18:1/18:0) was 76% specific and 66% sensitive as a biomarker	(60)

UPLC-MS/MS	CSF from 6 AD and 6 CN	↑ Gly, SAH, and ↓ SAM in AD CSF Established method for quantifying 17 metabolites of homocysteine-methionine metabolism	(61)

Biocrates Absolute IDQ p180 Kit measured by UPLC-MS/MS	732 fasting plasma samples from ADNI cohort	Bonferroni analysis correlated 13 metabolites with AD pathogenesis CSF Aβ42 metabolites: PC ae C36:2, PC ae 40:3, PC ae C42:4, PC ae C44:4, SM (OH) C14:1, SM C16:0 CSF t-tau/Aβ42 metabolites: C18, PC ae C36:2, SM C16:0, SM C20:2 Cognitive decline metabolites: C14:1, C16:1, SM C20:2, α-AAA, and Val Brain atrophy metabolites: C12, C16:1, PC ae C42:4, PC ae C44:4, α-AAA, and Val	(18)

HRMS	Plasma from 37 CN, 16 MCI, and 19 individuals who converted from MCI to AD (MCI_AD)	Polyamine and saturated fatty acid biosynthesis was most altered with MCI vs CN MCI_AD vs CN showed differences in cholesterol and sphingolipid transport and saturated fatty acid biosynthesis MCI_AD vs MCI was most perturbed in cholesterol and sphingolipid transport and polyamine metabolism Polyamine metabolism and l-Arg metabolism were common between CN, MCI, and MCI_AD	(62)

HPLC Lipidomics	Plasma from CN, MCI, and AD along with brain atrophy	10 molecules significantly altered that predicted AD patients with 79% accuracy including six ChEs following the trend CN > MCI > AD PC36:5 decreased in AD plasma associated with hippocampal atrophy Ceramides were associated with hippocampal atrophy in younger (age < 75 years) group while PCs correlated at age > 75 years	(63–65)

Biocrates Absolute IDQ p180 Kit by UPLC-MS	Plasma from 73 CN and 28 phenoconverters	Identified 24 plasma metabolites for the detection of preclinical AD with 95% accuracy 13 ↓ PCs (PC ae C34:0, PC ae C36:4, PC ae C40:6, PC ae C42:1, PC aa C32:0, PC aa C34:4, PC aa C36:6, PC aa C38:0, PC aa C38:3, PC aa C38:6, PC aa C40:1, PC aa C40:5, lysoPC a C18:2) 6 ↓ ACs (C3, C5, C5-OH (C3-DC-M), C9 C10:2, C18:1-OH) and 3 ↑ ACs (C10:1, C12:1, C16:2) Asn ADMA	(66, 67)

Biocrates Absolute IDQ p180 Kit measured by FIA-MS/MS and UPLC-MS	Plasma from 41 participants with superior memory, 109 CN, and 74 aMCI/AD	Developed a 12-metabolite panel for detection of superior memory valerylcarnitine, hydroxyhexadecadienylcarnitine, 3-hydroxypalmitoleylcarnitine, lysoPC a C28:1, lysoPC a C17:0, PC aa C38:5, Asp, Asn, Arg, histamine, citrulline, and nitrotyrosine	(68)

1H NMR	Saliva from 9 AD, 8 MCI, and 12 CN	Group separation achieved using logistic regression models Strongest predictive markers between MCI and CN were galactose, imidazole, and acetone with sensitivity and specificity of 90 and 94%, respectively	(69)

Faster UPLC-MS	Saliva from 256 AD and 218 CN	PCA identified sphinganine-1-phosphate, ornithine, phenyllactic acid, inosine, 3-dehydrocarnitine, and hypoxanthine as significantly altered in AD saliva ↑ sphinganine-1-phosphate in AD patients was a major biomarker with sensitivity of 99.4% and specificity of 98.2%	(70)

*1H NMR, proton nuclear magnetic resonance; AC, acylcarnitines where C denotes species; AD, Alzheimer’s disease; ADMA, asymmetric dimethylarginine; ADNI, Alzheimer’s Disease Neuroimaging Initiative; aMCI, amnestic MCI; Arg, arginine; ASYMAD, asymptomatic patients; Asn, asparagine; Asp, aspartate; ChE, cholesteryl ester; CN, control; CSF, cerebrospinal fluid; FIA-MS/MS, flow injection analysis-MS/MS; GC-MS, gas chromatography-MS; Gly, glycine; HILIC, hydrophilic interaction liquid chromatography; HPLC-MS/MS, high-performance liquid chromatography-MS/MS; HRMS, high-resolution MS; lysoPC, lysophosphatidylcholine; MCI, mild cognitive impairment; MS, mass spectrometry; MS/MS, tandem mass spectrometry; NAA, N-acetylaspartate; PC a, phosphatidylcholine acyl; PC aa, phosphatidylcholine diacyl; PC ae, phosphatidylcholine acyl-alkyl; PCA, principle component analysis; p-tau, phospho-tau; SAH, S-adenosylhomocysteine; SAM, S-adenosylmethionine; SM, sphingomyelin; t-tau, total-tau; UFA, unsaturated fatty acid; UPLC-MS/MS, ultra performance liquid chromatography-MS/MS; Val, valine; α-AAA, α-aminoadipic acid*.

## Metabolic Changes in Human Postmortem Brain Tissue and CSF

Using unbiased lipidomics and metabolomics approaches, Paglia and colleagues analyzed changes in postmortem frontal cortex from patients with AD and age- and sex-matched CN (58). Thirty four significantly altered metabolites that distinguished AD from CN belonged to six metabolic pathways: (1) alanine, aspartate, and glutamate metabolism, (2) arginine and proline metabolism, (3) cysteine and methionine metabolism, (4) glycine, serine, and threonine metabolism, (5) purine metabolism, and (6) pantothenate and CoA biosynthesis (58). Using partial least-squares regression, the authors correlated their metabolic findings with clinical significance (e.g., dementia and AD pathology). Results indicated that alanine, aspartate, and glutamate metabolism most strongly correlated with AD status while sex, age, body mass index, and postmortem interval had little to no correlation (58). Notably, their findings suggest that mitochondrial dysfunction, particularly aspartate metabolism, correlates with dementia and AD pathology. *N*-acetylaspartate (NAA) is a highly concentrated molecule in the brain, which is synthesized by mitochondria from aspartic acid and acetyl-CoA (71). Findings in AD patients showed a 15–20% reduction of NAA levels, which may relate to neuronal and mitochondrial dysfunction associated with decreased memory (71).

To identify brain region-specific metabolic changes, Snowden et al. used untargeted metabolomics to profile three brain regions differentially affected in AD patients (59). Brain tissue was collected from the cerebellum (CB), which is typically devoid of AD pathology, the middle frontal gyrus (MFG), and inferior temporal gyrus (ITG), which are vulnerable to Aβ and tau deposition, respectively. Furthermore, this study included individuals with AD, healthy CN, and asymptomatic patients (ASYMAD) without evident cognitive impairment but a significant display of AD pathology at death (59). Tissue samples from ASYMAD patients with AD pathology lacking dementia provide a unique longitudinal assessment. Additionally, observations made in brain regions differentially affected in AD enhance the understanding of spatial-temporal changes. The authors used untargeted metabolomics to measure 3,482 metabolites using LC-MS and an additional 1,415 metabolites using GC-MS. They identified six unsaturated fatty acids (UFAs; linoleic acid, linolenic acid, docosahexaenoic acid, eicosapentaenoic acid, oleic acid, and arachidonic acid) that correlated with AD pathology and clinical symptoms (59). The authors suggested that changes in the concentration of these UFAs may serve as regional threshold markers in the brain defining the onset of Aβ- and tau-induced cognitive decline (59). In the ITG, levels UFAs had the greatest change in ASYMAD when compared to CN. The MFG had a consistent alteration in UFA abundance with AD > ASYMAD > CN. Interestingly, the authors observed significant UFA changes in the CB of AD patients relative to CN suggesting that metabolic alterations are systemic affecting multiple brain regions irrelevant of the presence of Aβ and/or tau pathology.

Cerebrospinal fluid is the extracellular fluid that surrounds the brain representing an ideal source for determining neurobiochemical changes that occur in the central nervous system of AD patients. Using a Biocrates Absolute IDQ p180 metabolomics kit, which targets five compound classes (acylcarnitines, amino acids, biogenic amines, hexoses, and phospho- and sphingolipids), Koal et al. analyzed 50 CSF samples from patients with AD-like pathology defined by decreased Aβ42 and increased t-tau and p-tau CSF levels (60). Compared to healthy CN, they identified eight metabolites that were significantly increased in the CSF samples with AD-like pathology including one acylcarnitine (C3), two sphingomyelins [SM (d18:1/18:0) and SM (d18:1/18:1)], and five glycerophospholipids (PC aa C32:0, PC aa C34:1, PC aa C36:1, PC aa C38:4, and PC aa C38:6) (60). Using logistic regression analysis with forward variable selection, SM (d18:1/18:0) was identified as a potential biomarker capable of distinguishing between AD-like and healthy CN (60). Sphingomyelins represent major components of myelin sheaths. Catabolism of myelin and sphingomyelin has been shown to provide an alternative fuel source (in the form of ketones) in multiple diseases including AD and aging (27). Furthermore, sphingomyelin is enriched in lipid rafts that are also the site where gamma-secretases (PS1/PS2) localize, which may influence the processing of APP (72). While samples used in the study by Koal et al. were not derived from patients clinically diagnosed with AD, the concentration of SM (d18:1/18:0) significantly correlated with AD-like pathology as determined by levels of Aβ and tau in the CSF (60) suggesting that this marker may serve for early detection of the disorder.

In another study, Guiraud et al. utilized UPLC-MS/MS to quantitate 17 metabolites of the methionine cycle in the CSF of patients diagnosed with AD (61). They applied a new multianalyte strategy to monitor both metabolites and cofactors of methionine metabolism simultaneously. Using this approach, Guiraud and colleagues identified significant increases of glycine and *S*-adenosylhomocysteine (SAH) with decreased *S*-adenosylmethionine (SAM) in the CSF of patients diagnosed with AD (61). The metabolites SAM and SAH are key intermediates in the methionine cycle important for protein synthesis and maintaining cellular methylation of DNA, proteins, and neurotransmitters (73). In cells, methionine is primed forming SAM, which serves as a cofactor for methyl transferases. SAH is generated after transfer of the methyl group is complete. Dysregulation of methionine, SAM, and SAH metabolism has been linked to several neurodegenerative diseases including AD (74–76). For example, mice fed with an l-methionine-enriched diet had increased levels of Aβ oligomers and p-tau (77). Additionally, several studies have demonstrated a strong correlation between AD and altered DNA methylation profiles (78–80). Combined, these findings provide insight into possible epigenetic alterations that may explain the sporadic nature of AD.

## Metabolic Profiling in Human Plasma

Plasma is an easily accessible biofluid suitable for recurrent measures. Multiple studies were conducted in the plasma of patients with different severity of AD to establish metabolic changes indicative of the disease progression (57). Our earlier studies demonstrated that changes in metabolic pathways involved in altered energy homeostasis detected in the CSF of patients with MCI and AD could be accurately recapitulated in plasma (30). However, the reproducibility of results reported in a number of studies using metabolic profiling in plasma varied substantially (57). Part of the problem includes small sample sizes and inconsistent processing techniques. One of the largest metabolomics studies to date was conducted in plasma from AD patients collected within the ADNI cohort by researchers of the Alzheimer’s Disease Metabolomics Consortium (18). In this study, Toledo et al. analyzed serum from over 700 ADNI participants and correlated metabolic changes with clinical data available for each patient including CSF levels of Aβ42 and tau, brain structure assessed by MRI, and cognitive performance determined by ADAS-Cog scores (18, 81–83). Using Bonferroni multiple comparisons, Toledo et al. identified 13 key metabolites altered at the various stages of AD. Six metabolites were associated with increased CSF Aβ42 including PC ae C36:2, PC ae C40:3, PC ae C42:4, PC ae C44:4, SM (OH) C14:1, and SM C16:0, and four were linked with t-tau/Aβ42 ratio (C18, PC ae C36:2, SM C16:0, SM C20:2). Five metabolites (C14:1, C16:1, SM C20:2, α-aminoadipic acid [α-AAA], and valine) accompanied decreased cognitive function while six (C12, C16:1, PC ae C42:4, PC ae C44:4, α-AAA, and valine) correlated with increased brain atrophy (18). For each of the clinical predictors of AD, a decrease in valine and α-AAA were detected while levels of acylcarnitines, PCs, and SMs increased. The integrated approaches taken by Toledo and colleagues provided valuable insight into mechanisms of AD (18). First, they found that changes in the membrane lipid PCs and SMs occurred in the initial stages of AD pathology associated with abnormal CSF Aβ42 levels, which may suggest that membrane alterations involved in neurodegeneration begin early (18). Following aberrant Aβ levels, the authors identified tau-associated changes in long-chain acylcarnitines and SMs implicated in lipid metabolism. Since tau-associated metabolite alterations preceded brain atrophy and cognitive decline and were distinctive from Aβ42 perturbations, these results may indicate intermediate changes that correlate with altered lipid metabolism and mitochondrial bioenergetics (18). This further suggests that tau-associated metabolic alterations could serve as an intermediate biomarker indicative of cognitive decline. Lastly, partial correlation networks and coexpression network analysis suggested that changes in brain volume and cognition (assessed by MRI and ADAS-Cog scores) correlated with a shift in substrate utilization from fatty acids to amino acids (18). This AD-associated shift in energy metabolites provides new insights into metabolic transitions that occur late in the disease process. Amino acids, particularly alanine and glutamine in addition to threonine, glycine, and serine, can be used to synthesize glucose *via* gluconeogenesis (84). As the brain is highly glycolytic, this may indicate an attempt to reestablish glycolysis that is altered in AD (Figure 1). Taken together, this study suggests that specific metabolic changes occur during the progression of AD and that blood-based metabolite markers could improve disease diagnosis.

In line with results from Toledo et al. (18), several additional metabolomics studies have revealed perturbed metabolic profiles in the plasma of AD patients. Graham and colleagues (62) used high-resolution MS in a longitudinal study to analyze metabolic changes in plasma samples from 37 healthy CN, 16 patients with MCI, and 19 patients that converted from MCI to AD (MCI_AD). Comparison of MCI to CN showed that polyamine metabolism and saturated fatty acid biosynthesis were highly altered. The most affected metabolic pathways when comparing MCI_AD to CN included cholesterol and sphingolipid transport and saturated fatty acid biosynthesis. Conversion from MCI to MCI_AD was associated with metabolic alterations in cholesterol and sphingolipid transport and polyamine metabolism. Consistently disrupted across all three comparisons, however, was polyamine metabolism and l-arginine metabolism suggesting that these metabolic pathways are highly implicated in the conversion of healthy individuals to MCI and AD (62). The authors further noted that several metabolites were shared across polyamine and l-arginine metabolism, which have been reported to be important for neuronal health, survival, growth, and cell death (62, 85–89). Polyamine synthesis begins in the mitochondria *via* conversion of arginine to ornithine by the enzyme arginase (ARG2) (90). Subsequent reactions lead to the production of polyamines (e.g., spermidine and spermine). Of note, methionine/SAM metabolism is required for polyamine synthesis, which was found to be significantly increased in the CSF of AD patients by Guiraud and colleagues (61, 90). The polyamines spermine and spermidine have also been shown to act as agonists for *N*-methyl-d-aspartate (NMDA) receptors that bind to the neurotransmitter glutamate (91). The drug memantine, approved for the treatment of AD, is an NMDA antagonist, which reduces excitotoxicity of glutamate signaling protecting against synaptic loss and cognitive decline (92). Metabolic findings by Graham and colleagues suggest a role for polyamines in NMDA-mediated excitotoxicity in AD.

Proitsi el al. used untargeted lipidomics to compare plasma from 123 individuals (40 CN, 48 MCI, and 35 AD patients from the Dementia Case Register cohort and EU funded AddNeuroMed study) (63). Using univariate analysis, Proitsi et al. identified 41 metabolites associated with AD. After applying Random Forest and backward elimination, the authors narrowed the list of metabolites to a panel of 10 molecules that could identify AD patients with 79% accuracy. Six of these metabolites were identified as cholesteryl esters (ChEs), which Proitsi et al. noted as a class of metabolites not previously associated with AD (63). However, previous findings have reported alterations of phosphatidylcholines (PCs) in AD (93, 94), which are precursors for the synthesis of ChEs. The enzyme lecithin-cholesterol acyltransferase synthesizes ChEs from PC and cholesterol to allow for more efficient transport of cholesterol in the blood stream. In two recent follow-up studies from Legido-Quigley’s group, the authors further implicated changes in plasma lipids in AD. Kim et al. demonstrated a correlation between altered lipid concentrations in AD patients and brain atrophy (64). Three ceramides (Cer16:0, Cer18:0, and Cer20:0) were elevated in the plasma of AD patients and were associated with hippocampal atrophy in adults younger than 75 years of age. For AD patients over 75 years of age, a decrease in PC38:6 and PC40:6 were linked to hippocampal atrophy. These findings suggest that impaired PC and ceramide metabolism could be associated with various stages of AD progression and hippocampal atrophy (64). Using additional samples from the Dementia Case Register cohort and EU funded AddNeuroMed study (148 AD and 152 CN), Proitsi et al. replicated their previous findings in the plasma of MCI and AD patients (63, 65). Proitsi further demonstrated a correlation between altered lipids (including new putative lipids) with AD progression and brain atrophy (65). Together, the results from Legido-Quigley’s group support an association between altered plasma PCs, ChEs, and ceramides with AD progression, which is consistent with the implication of altered lipid metabolism in AD pathogenesis (27, 30, 95–97).

Mapstone and colleagues previously developed a panel of blood-based biomarkers that could predict the conversion of cognitively normal individuals to amnestic MCI (aMCI) or AD, referred to as phenoconversion (66). Their panel consisted of 10 plasma lipids, which could predict phenoconversion of individuals (approximately 80 years of age) within a 2–3-year timeframe with 85% accuracy (66). However, low positive predictive values remained an issue reducing clinical value. More recently, using the same cohort, Fiandaca et al. refined and expanded the blood-based panel to include 24 metabolites resulting in increased positive predictive values (67). The updated panel consists of 13 glycerophosphatidylcholines (PCs), 9 acylcarnitines, asparagine, and asymmetric dimethylarginine (ADMA), which improved their predictive accuracy to 95% (67). These results are in line with other studies where plasma PCs, acylcarnitines, and amino acids were found to be altered in MCI and AD patients (18, 62–65). Additionally, two metabolomics studies using CSF or brain tissue from patients without cognitive impairment but with AD-like pathology also identified differentially regulated lipids and UFAs when compared to CN (59, 60). In the CSF of AD patients, Guiraud et al. observed significant changes in SAM (61), which is needed for the generation of ADMA (98). These results strongly implicate lipids as pre- and post-symptomatic markers for dementia and AD. Furthermore, these data strengthen the notion that peripheral blood may be an appropriate biofluid that can reflect changes in the CSF and brain tissue.

Aging is characterized by a decline in memory and cognition and is considered the greatest risk factor for AD. In search for metabolic markers of healthy aging, Mapstone et al. identified metabolites in older individuals (approximately 80–85 years of age) with superior memory performance compared to normal and aMCI/AD patients (68). Participants were scored based on cognitive performance including attention, executive, language, memory, and visuospatial properties. Performance studies identified 41 participants with superior memory, 109 with normal memory performance, and 74 people were classified as having aMCI/AD. Plasma samples were analyzed using the Biocrates Absolute IDQ p180 Kit measured with FIA-MS/MS and UPLC-MS. Comparison of individuals with superior memory to controls by least absolute shrinkage selection operator analysis revealed 12 metabolites capable of distinguishing between the two groups. The 12-metabolite panel included valerylcarnitine, hydroxyhexadecadienylcarnitine, 3-hydroxypalmitoleylcarnitine, lysoPC a C28:1, lysoPC a C17:0, PC aa C38:5, aspartate, asparagine, arginine, histamine, citrulline, and nitrotyrosine (68). Using this panel, the authors were able to distinguish between the elderly with superior memory and aMCI/AD or preclinical AD suggesting these markers may indicate early memory deficits (68). Interestingly, their previously identified 10-lipid panel (66), which putatively detected early signs of neurodegeneration, only modestly associated with the 12-metabolite healthy aging panel (68). Superior memory-associated metabolites identified by Mapstone and colleagues may provide insight into metabolic pathways that are important for proper cognitive function.

## Metabolic Profiling in Human Saliva

Saliva is a readily accessible biofluid that contains proteins, mRNA, microRNA, enzymes for the breakdown of lipids and starches, and molecules important for biological functions including taste, lubrication, and immune responses (99–101). Unlike CSF and plasma, composition of saliva rapidly changes in response to biological stimuli (102, 103). In a recent study, Yilmaz et al. utilized NMR-based metabolomics to identify 22 salivary metabolites that were useful for distinguishing between AD, MCI, and healthy patients (69). Group separation between AD and CN was achieved using logistic regression models identifying significant changes in the metabolites propionate and acetone (69). Similarly, galactose, imidazole, and acetone distinguished between MCI and CN, while creatine and 5-aminopentanoate separated AD vs MCI (69). In another study using faster UPLC-MS, the authors analyzed the saliva from 256 patients with AD and 218 age-matched healthy controls (70). Using principal component analysis, Liang and colleagues identified six metabolites (Sphinganine-1-phosphate, ornithine, phenyllactic acid, inosine, 3-dehydrocarnitine, and hypoxanthine) that were significantly different in AD patients compared to CN. Three of these metabolites (sphinganine-1-phosphate, ornithine, and phenyllactic acid) were strong predictors of AD (predictive accuracy; area under curve = 0.998) (70). Ornithine is an intermediate metabolite of polyamine metabolism, which was affected in the plasma of AD patients (62). These results demonstrate the potential use of metabolomics to develop salivary biomarkers capable of diagnosing AD.

## Metabolomics in Mouse Models of AD

In 1907, Dr. Alois Alzheimer described the first patient with senile plaques and NFTs, which represent the major hallmarks of AD (104, 105). Since the initial description, significant progress has been made to enhance our understanding of AD mechanisms including the identification of familial gene mutations in APP (106–108), PS1, and PS2 (109, 110) implicated in the accumulation of amyloid peptides. While only a very small number of AD cases are associated with familial mutations, the development of mouse models that express human APP, PS1, and PS2 transgenes significantly expanded the ability to study disease mechanisms. Mice can also harbor the human transgene encoding MAPT, which in its hyperphosphorylated state forms NFTs (111). Currently, along with the popular models of FAD including APP (Tg2576), APP/PS1, or 3xTg AD mice, the development of humanized mouse models expressing genetic risk factors, such as APOE ε4 allele, allows researchers to study mechanisms of late-onset sporadic AD (111–113). Table 3 represents some of the most commonly used mouse models of AD.

**Table 3:** Common transgenic mouse models of AD utilized in metabolomics studies.

Model	Transgene/mutation	Phenotype	Reference
APP (Tg2576)	APP: KM670/671NL (Swedish)	5- and 14-fold increase of Aβ_40_ and Aβ_42/43_, respectively Aβ plaques by 11 months Gliosis identified near Aβ plaques by 10 months Cognitive impairment detected by 3–6 months	(114, 115)

PS1 (line 5.1)	PSEN1: M146L	2- to 3-fold increase of mutant PSEN1 Elevated Aβ_42/43_ in the brain	(116)

APP/PS1	APP: KM670/671NL (Swedish) PSEN1: M146L	Enhanced pathology compared to single transgene Aβ deposits by 6 months Gliosis by 6 months Cognitive impairment detected by 3 months	(117, 118)

3xTg	APP: KM670/671NL (Swedish) PSEN1: M146V MAPT: P301L	Age-associated pathology Aβ deposits by 6 months Tau pathology by 12 months Gliosis by 7 months Cognitive impairment detected by 4 months	(119–121)

5xFAD	APP: KM670/671NL (Swedish); I716V (Florida); V717I (London) PSEN1: M146L; L286V	Early and aggressive presentation Aβ deposits by 1.5 months Gliosis by 2 months Cognitive impairment detected by 4 months	(122, 123)

APOE4	APOE4 targeted replacement	APOE levels and plasma lipids in ε4 mice do not differ significantly to ε3 mice APOE4 mice have reduced VLDL clearance rate compared to APOE3 mice	(124)

EFAD	5xFAD with APOE (2, 3, or 4) knock-in	APOE4 mice (E4FAD) have increased plaques compared to E3FAD and E2FAD models Plaque formation between 4 and 6 months Gliosis at 6 months of age in all models Cognitive decline in E4FAD > E3/E2FAD	(125)

*APOE, apolipoprotein E; APP, amyloid precursor protein; FAD, familial Alzheimer’s disease; MAPT, microtubule-associated protein tau; PS1/PSEN1, presenilin-1; VLDL, very low density lipoprotein*.

Mice have become a common model to study AD since they share 99% of their genes with humans, have a relatively short life span (approximately 2–3 years), are easy to handle and to house, have a simple reproductive scheme, and can be genetically modified (126). However, while mice carry endogenous genes encoding for APP and tau, they do not naturally develop AD. While the exact reasons for that are unknown, it may be due to the three amino acid difference between human and rodent Aβ or that mice do not live long enough (127). Although there is no single mouse model that recapitulates the complexity of human AD, transgenic mice provide an invaluable resource for studying the effect of individual genetic components on the disease progression and for preclinical validation of experimental therapeutics (114–121). Application of metabolomics confirmed early energetic and metabolic alterations in multiple mouse models of FAD that recapitulate human conditions (33, 57). The most recent applications of metabolomics to study metabolic disturbances in biofluids and brain tissue of AD mouse models are summarized in Table 4.

**Table 4:** Application of metabolomics in samples from AD mouse models.

Analytical platform	Samples	Findings	Reference
DIMS	Brains and plasma from APP/PS1 and WT mice	APP/PS1 cortex and hippocampus had altered phospholipids and ACs APP/PS1 blood serum had significant alterations in eicosanoids (LB4, HEPE, and prostaglandins) Studies suggest altered lipid metabolism and energy utilization in APP/PS1 mice	(128, 129)

Absolute IDQ p180 Kit measured by UPLC-MS	Longitudinal collection (6–18 months) of APP/PS1 and WT mouse brains and plasma	6 months: ↑ Arg in brain, ↓ Gln and Pro in plasma At 6–10 months: ↑ polyamines putrescine, spermidine, and spermine in brain and plasma 10–12 months: ↓ Thr 12 months: ↓ Gln and citrulline in plasma Potential temporal disturbance in amino acids and lipid metabolism	(130)

Bile acid kit measured by LC-MS/MS	Plasma and whole brain tissue from 5 APP/PS1 at 6 and 12 months of age Plasma and neocortex from 10 AD and CN patients	Bile acids are perturbed in AD samples Human plasma had ↓ CA in AD patients APP/PS1 mouse plasma had ↑ CA at 6 months and ↓ hyodeoxycholic acid at 12 months Human neocortex had ↓ taurocholic acid APP/PS1 brain tissue: 6 months had ↑ lithocholic acid and ↓ TMCA; 12 months had ↓ TMCA, CA, β-muricholic acid, Ω-muricholic acid, taurocholic acid, and tauroursodeoxycholic acid	(131)

UHPLC-MS	Urine from 30 APP/PS1 and CN mice at 2 months of age	Identification of potential early biomarkers in urine ↑ Spermic acid, 2,4-guanidinobutanoic acid, nicotinuric acid, l-isoleucyl-l-proline, l-2,3-dihydrodipicolinate, 3,4-dihydroxyphenylglycol o-sulfate, *N*-acetyl-l-tyrosine, 5-hydroxyindoleacetic acid, 3-methoxybenzenepropanoic acid, and 3,4-dimethoxyphenylacetic acid Dimethylarginine, 1-methyladenosine, citric acid, 5′-deoxyadenosine, 1-(beta-d-ribofuranosyl)-1,4-dihydronicotinamide, 7-methylinosine, 2-keto-6-acetamidocaproate, 7-aminomethyl-7-carbaguanine, succinyladenosine, benzaldehyde, urothion, 6-hydroxy-5-methoxyindole glucuronide, monobutyl phthalate, and tetrahydrocortisol Had greatest impact on glyoxylate and dicarboxylate metabolism	(132)

Head-space GC-MS	Urine of 15 APP mice, 15 Tg2576 mice, 9 TgCRND8 mice, and 10 APPLd2 mice and NTG littermates	↑ Phenylacetone across all three APP mice Linear discriminant analysis predicted groups with <16% error Predictive metabolites include 6-hydroxy-6-methyl-3-heptanone, 3-methylcyclopentanone, 4-methyl-6-hepten-3-one, 1-octen-3-ol, 2-sec-butyl-4,5-dihydrothiazole, acetophenone, phenylacetone, o-toluidine	(133)

LC-MS and GC-MS	Cortex and plasma from symptomatic APP/PS1 mice	CAD-31 was found to be neuroprotective CAD-31 in plasma of APP/PS1 mice ↑ sphingolipids (glycosyl-*N*-stearoyl-sphingosine and sphingosine-1-phosphate) CAD-31 in cortex of APP/PS1 mice ↑ monoacylglycerols (1-palmitoylglycerol, 2-palmitoylglycerol, 2-oleoylglycerol) CAD-31 in plasma of control mice ↓ long-chain fatty acids (margarate, pentadecanoate, 10-nonadoconoate), ↑ acylcarnitines (C0, C16, C18:1), ↑ ketone body 3-hydroxybutyrate, ↑ sphingolipids (glycosyl-*N*-stearoyl-sphingosine, sphinganine-1-phosphate, sphingosine-1-phosphate, sphinganine) CAD-31 in cortex of control mice was similar to plasma	(134)

HPLC-QTOF-MS	Plasma from AD-induced mice (*via* Aβ42 injection) and controls (n = 8 per group)	Breviscapine treatment was neuroprotective in Aβ injected mice Multivariate analysis of breviscapine treated Aβ mice identified indoleacrylic acid, C16 sphinganine, LPE (22:6), sulfolithocholic acid, LPC (16:0), PA (22:1/0:0), taurodeoxycholic acid, and PC (0:0/18:0) Phospholipid and cholesterol modulation may be neuroprotective	(135)

IC-MS/MS	Primary astrocytes of 5xFAD mice from neocorticies of 1- to 3-day-old mice	Pantethine has anti-inflammatory properties AD astrocytes treated with pantethine had improved glycolytic and TCA cycle flux Pantethine treatment in AD astrocytes augmented glucose-6-phosphate, glycerol-3-phosphate, αKG, fumarate, and succinate levels	(136)

EIS-MS/MS	BMDMs derived from Trem2^−/−^ and WT mice	↓ UDP-glucose, CDP-ethanolamine, glucose-6-phosphate, fructose bisphosphate, citrate, and succinate ↑ Indolacetate, glycerol-3-phosphate, malate, and fumarate TREM2 deficiency perturbs mTOR signaling and nucleotide, glycolytic, and TCA cycle metabolites Cyclone creatine supplement alleviates TREM2 deficiency in BMDMs	(137)

*αKG, αketoglutarate; AD, Alzheimer’s disease; Arg, arginine; APP, amyloid precursor protein; BMDMs, bone marrow-derived macrophages; CA, cholic acid; CDP, cytidine diphosphate; CN, control; DIMS, direct infusion mass spectrometry; EIS-MS/MS, electrospray ionization; FAD, familial Alzheimer’s disease; Gln, glutamine; GC-MS, gas chromatography-MS; HEPE, hydroxy-eicosapentaenoic acid; HPLC-QTOF-MS, high-performance liquid chromatography quadropole-time-of-flight MS; IC-MS/MS, ion chromatography-MS/MS; LB4, leukotriene B4; LC-MS, liquid chromatography-MS; LPC, lysophosphatidylcholine; LPE, lysophosphatidylethanolamine; MS/MS, tandem mass spectrometry; mTOR, mammalian target of rapamycin; NTG, non-transgenic; PA, phosphatidic acid; PC, phosphatidylcholine; Pro, proline; PS1/PSEN1, presenilin-1; TCA, tricarboxylic acid; Thr, threonine; TMCA, tauromuricholic acid; TREM2, triggering receptor expressed on myeloid cells 2; UDP, uridine diphosphate; UHPLC, Ultra high-performance liquid chromatography; UPLC-MS/MS, ultra performance liquid chromatography-MS/MS; WT, wild type*.

## Metabolic Profiling in Brain Tissue and Blood from Transgenic Mouse Models of AD

In two consecutive studies, Gonzalez-Dominguez and colleagues used direct infusion MS (DIMS) to explore altered metabolic profiles in APP/PS1 mice compared to non-transgenic (NTG) CN mice (128, 129). In the first study, the authors focused on the evaluation of region-specific metabolic changes in the hippocampus, cortex, CB, and olfactory bulbs of male and female mice 6 months of age. The greatest changes in metabolite composition were found in the cortex and hippocampus of AD compared to NTG mice including the accumulation of fatty acids and alterations in phospholipids and acylcarnitines related to neural membrane degradation and impaired β-oxidation, respectively (128). In all brain regions examined, levels of the neurotransmitter dopamine were altered in AD compared to NTG mice similar to that observed in AD patients (128, 138, 139). The cortex and hippocampus are two regions primarily affected in AD with high levels of amyloid plaques and NFTs (140). Consistent with this, the authors found the greatest disturbances in the cortex and hippocampus supporting that metabolic alterations are closely linked to neuropathological changes in AD (141, 142). Similar to AD patients, region-specific changes in fatty acids were detected in postmortem AD brains (59).

In the second study, Gonzalez-Dominguez and colleagues analyzed serum from APP/PS1 mice using a two-step extraction method to increase the range of metabolites detected (129). Combined DIMS with electrospray ionization and flow injection atmospheric pressure photoionization MS techniques allowed for a fast comprehensive scan of polar and non-polar metabolites, respectively. The largest alterations in the serum of APP/PS1 compared to NTG mice were found in levels of eicosanoids including leukotriene B4 (LTB4), hydroxy-eicosapentaenoic acid (HEPE), and prostaglandins (129). Eicosanoids are derived from polyunsaturated fatty acids known for their role in inflammatory and immune responses (143). Consistent with these results, eicosanoids have been linked to neuroinflammation and AD etiology (144, 145). Metabolomics studies in human plasma also identified perturbed fatty acid metabolism (18) further suggesting that APP/PS1 mice recapitulate peripheral metabolic changes associated with the progression of AD in humans.

One of the advantages for studying AD in transgenic mouse models is the ability to easily collect and analyze longitudinal samples for metabolic changes during various disease stages in respect to amyloid deposition and cognitive function. Pan et al. conducted one of the first longitudinal metabolic profiles using brain tissue and plasma from 6, 8, 10, 12, and 18 months old APP/PS1 mice relative to NTG controls (130). Longitudinal samples were analyzed using a Biocrates Absolute IDQ p180 Kit measured by UPLC-MS. Metabolic pathway analysis identified perturbed polyamine metabolism in the brain tissue and plasma of APP/PS1 mice. Additional alterations were detected in essential amino acids, branched-chain amino acids, and the neurotransmitter serotonin. The authors noted that metabolic changes detected in the brain tissue of APP/PS1 mice were observed in the blood 2–4 months later (130). For instance, phospholipids were found most significantly altered in the brain of APP/PS1 mice at 8 months of age with the same alterations detected in blood 4 months later. This suggests a temporal sequence of events where changes in brain metabolites precede those in the blood. The authors determined that group separation between APP/PS1 and NTG mice based on metabolic signatures was the most pronounced between the ages of 8 and 12 months. In contrast, the brain and blood metabolic profiles between APP/PS1 and NTG mice in the youngest (6 months) and oldest (18 months) populations were less clearly separated (130). In the study by Pan and colleagues (130), it is worth noting that over a span of 12 months, the metabolic disturbances in APP/PS1 mice were transient suggesting that increasing AD pathology has a progressive impact on metabolism. This is in agreement with observations reported by Toledo et al. in the plasma of patients with AD enrolled in the ADNI study (18). Furthermore, Pan et al. detected increased levels of the polyamines spermine and spermidine at 8 months in the brain tissue and plasma of APP/PS1 mice (130), which is similar to changes identified in our previous study (30) and by others in the plasma of AD patients (62).

## Metabolic Profiling in Mouse and Human Bile Acids

Primary bile acids are synthesized in the liver and are well known for their role in cholesterol catabolism (146). Bile acid-induced signaling through nuclear receptors and cell surface G-proteins can modulate several metabolic, immune, and inflammatory processes (147). Secretion of bile acids into the intestine results in further modification by gut microbiota, which is thought to influence brain morphology, injury, and stress *via* the gut–brain axis (148). Increasing evidence implicates bile acids in several neurological diseases including AD (149–151). Feeding APP/PS1 mice with 0.4% tauroursodeoxycholic acid for 6 months resulted in decreased levels of Aβ in the hippocampus and cortex along with increased memory performance (152). Furthermore, therapeutic approaches targeting bile acid metabolism and signaling have shown beneficial effects in several metabolic and neurodegenerative disorders (151, 153–155). Using human and mouse samples, Pan et al. investigated longitudinal changes of bile acids in respect to AD development (131). Metabolic changes in human postmortem brain tissue (neocortex, *n* = 10) from confirmed AD cases along with plasma obtained from patients predicted to have AD (*n* = 10) was compared to age-matched controls. Additionally, the authors profiled whole brain tissue and plasma from APP/PS1 mice at 6 and 12 months of age. The authors quantitated 22 bile acids using the Biocrates Bile Acid Kit analyzed by LC-MS/MS (131). In human plasma, they identified a single bile acid, cholic acid, which was significantly lower in AD patients compared to controls. Analysis of AD brain tissue from humans revealed a significant decrease in taurocholic acid (131). Bile acid analysis in the plasma from APP/PS1 mice revealed that cholic acid was significantly higher in 6-month-old mice while hyodeoxycholic acid was decreased in 12-month-old mice compared to NTG controls (131). In APP/PS1 mouse brain tissue, the authors identified two perturbed bile acids at 6 months of age compared to NTG. At 12 months of age, APP/PS1 mice had six bile acids that were significantly altered. Among the altered bile acids, only tauromuricholic acid was decreased in APP/PS1 mice in both age groups (131). A decrease in taurocholic acid was observed in both human AD cortex and APP/PS1 mouse brain tissue (131). Hydrolysis of taurocholic acid yields the nonessential amino acid taurine (156). Transgenic APP/PS1 mice administered taurine *via* drinking water displayed improved cognition with slightly reduced levels of Aβ in the cortex (157). Taken together, these findings warrant further investigation into the relationship between bile acid perturbations and AD.

## Metabolic Profiling in Mouse Urine

Yu et al. investigated metabolic changes in the urine of APP/PS1 transgenic mice prior to cognitive impairment (132). At 2 months of age, the spatial working memory of APP/PS1 mice showed no significant differences when compared to NTG controls (132). However, metabolomics analysis of urine from the 2-month-old mice indicated that several metabolites were differentially regulated in APP/PS1 mice compared to controls. Applying UPLC coupled with quadruple time-of-flight MS, Yu et al. identified 10 metabolites that were significantly upregulated and 14 metabolites that were downregulated in APP/PS1 mice (132). These results suggest that early metabolic changes occur prior to spatial memory and cognitive impairment. Pathway analysis of the dysregulated metabolites allude to perturbations in pentose and glucoronate interconversions, glyoxylate and dicarboxylate metabolism, starch and sucrose metabolism, the citrate cycle, arginine and proline metabolism, and tryptophan metabolism early in APP/PS1 mice compared to controls (132). The metabolite 5-hydroxyindoleacetic acid (5-HIAA) was significantly increased in the urine of APP/PS1 mice compared to controls (132). In urine, the concentration of 5-HIAA is routinely used to estimate levels of serotonin in the body (158). Serotonin is a neurotransmitter that can affect cognition, and changes in its levels have been implicated in AD (31, 159, 160). Therefore, metabolic analysis of urine from APP/PS1 mice may reflect fluctuations in the brain.

Using GC-MS, levels of volatile metabolites were investigated in the urine from three different transgenic APP mouse models (133). Each mouse model has a distinct pathological time course (114, 161, 162). The three strains included the Tg2576, TgCRND8, and APPld2 mice, which developed amyloidosis between 3–16, 2–8, and 4–23 months of age, respectively (133). The authors identified seven urine metabolites that discriminated transgenic mice from NTG littermates. Common across all three transgenic models was a disruption in 1-octen-3-ol (octenol) (133), which has previously been implicated in disrupting dopamine homeostasis resulting in neurodegeneration (163). Three additional unique metabolites were identified in Tg2576 mice (2-sec-butyl-4,5-dihydrothiazole, acetophenone, and phenylacetone), one in TgCRND8 mice (4-methyl-6-hepten-3-one), and two in APPLd2 mice (3-methylcyclopentanone and o-toluidine) (133). While these results identified a common dysregulated metabolite (octenol) in the urine of all three transgenic mouse models, their respective unique signatures further suggest that varying Aβ pathology has a gradual impact on the metabolome.

## Application of Metabolomics to Monitor Therapeutic Efficacy

To date, there are no disease-modifying strategies for AD. Multiple clinical trials designed to reduce Aβ production or clearance have failed (164). One explanation is that most treatments were administered at a late stage of the disease when irreversible damage has already occurred. There is a clear need for more accurate and early diagnosis of people who are on the trajectory to develop AD so clinical trials can be conducted in individuals where the course of the disease could be modified or reversed. Additionally, there is a need to monitor therapeutic efficacy of experimental treatments. While some clinical trials now include the collection of biofluids to monitor therapeutic outcomes using metabolomics, these results have not been published yet. Several recent studies, however, have used metabolomics to monitor efficacy of new therapeutic approaches in preclinical transgenic mouse models of AD.

Schubert and colleagues developed a drug that has neurogenic and neuroprotective properties (134, 165, 166). The drug derives from a hybrid of curcumin and cyclohexyl-bisphenol A (166, 167). A derivative of this compound, CAD-31, showed potent neuroprotection and memory enhancing properties in AD mouse models (165). In a follow-up study, Daugherty and colleagues studied the effects CAD-31 on the metabolome of AD mice (134). Transgenic female APP/PS1 mice were administered CAD-31 starting at 10 months of age for 3 months. Cortex brain tissue and plasma samples from CAD-31 treated and untreated mice were analyzed using non-targeted GC-MS and LC-MS (134). Metabolomics analysis in plasma of AD mice demonstrated that sphingolipids were significantly altered by CAD-31 treatment, while ketone bodies, long-chain fatty acids, acylcarnitines, and sphingolipids were affected in CN mice (134). In the cortex of CAD-31-treated APP/PS1 mice, the most significantly altered metabolites were monoacylglycerols including 1- and 2-palmitoylglycerol and 2-oleoylglycerol (134). Decreased brain glucose utilization is a well-characterized early metabolic phenotype detected in AD patients. This brain hypometabolism indicates the requirement for the use of alternative fuel sources in order to maintain neuronal function (27, 168, 169). The neuroprotective and memory enhancing effects seen in AD mice treated with CAD-31 may, in part, be associated with a metabolic shift toward lipid utilization. Metabolites affected by CAD-31 treatment in WT mice included ketones, acylcarnitines, and acetyl-CoA, which suggest that mitochondrial energetics and lipid metabolism were favorably altered. However, in APP/PS1 mice, only monoacylglycerols were significantly altered by CAD-31 treatment (134). The monoacylglycerol 2-arachidonoylglycerol (2-AG), known for its role in neurotransmission (170), has previously been implicated in the pathogenesis of AD (171). Hydrolysis of 2-AG by monoacylglycerol lipase results in the formation of arachidonic acid, which is a precursor for the eicosanoids LTB4, HEPE, and prostaglandins (171–174). In line with these findings, Gonzalez-Dominguez and colleagues identified altered levels of LTB4, HEPE, and prostaglandins in the serum of APP/PS1 mice (129). These findings could indicate that modulation of lipid metabolism and monoacylglycerols may be an effective therapeutic strategy to favorably alter cellular energetics and synaptic signaling in AD.

Erigerontis Herba is a traditional Chinese medicine used to treat cardiovascular diseases (175). Scutellarin, a flavone found in Erigerontis Herba, has been shown to prevent β-amyloid aggregation (176). In an attempt to identify the underlying therapeutic mechanisms of Erigerontis Herba administration, Xia et al. investigated metabolic changes in AD mice treated with breviscapine, a drug containing >85% scutellarin (135, 175). In this study, AD was induced in mice *via* unilateral ventricle injection of aggregated Aβ42 peptides. AD-induced mice displayed decreased learning and memory abilities compared to controls (135). Dose-dependent treatment of AD mice treated with breviscapine showed improved performance in behavior tests compared to untreated controls (135). Plasma from AD mice treated with breviscapine was analyzed using LC-MS metabolomics. The authors identified eight metabolites implicated in lipid metabolism including indoleacrylic acid, C16 sphinganine, lysophosphatidylethanolamine, sulfolithocholic acid, lysophosphatidylcholine, phosphatidic acid, taurodeoxycholic acid, and phosphatidylcholine (135). Consistent with other studies (66, 67, 131), these results suggest that modulation of lipid metabolism in AD can enhance neuroprotection.

Unbalanced inflammatory mechanisms from several neural cells, including microglia, oligodendrocytes, and astrocytes, are thought to contribute to the progression of AD (177). Pantethine, a precursor to vitamin B5, has previously been shown to alleviate symptoms related to immune and inflammatory responses (178, 179). Furthermore, pantethine has been shown to be beneficial in treating metabolic disorders associated with mitochondrial dysfunction and neurodegeneration (180–182). Using astrocytes from newborn 5xFAD mice, van Gijsel-Bonnello et al. studied early mechanisms of inflammation and metabolic disorder that contribute to AD (136). The authors evaluated if treatment with pantethine in 5xFAD astrocytes would prevent inflammatory and metabolic dysfunction (136). Non-targeted analysis revealed disturbances in metabolites of the glycolytic pathway (glucose-6-phosphate and glycerol-3-phosphate) and TCA cycle (αketoglutarate, fumarate, and succinate) in 5xFAD astrocytes compared to controls (136). Pantethine treatment reduced the extent of alterations seen in 5xFAD astrocytes (136). The authors linked improved glycolytic and TCA cycle flux with nucleotide homeostasis and increased ATP levels (136). Additionally, they reported reduced inflammatory processes in 5xFAD astrocytes treated with pantethine highlighting the benefits of augmenting altered brain energetics early in AD (136).

The protein triggering receptor expressed on myeloid cells 2 (TREM2) is a cell surface receptor known for its role in immune signaling (183). Recent GWAS studies associated a mutation in TREM2 (R47H) with increased risk for AD (184). TREM2 was further shown to be a sensor for lipids associated with Aβ, which is impaired by the R47H mutation (185). Furthermore, Trem2 deficiency in 5xFAD mice led to increased Aβ burden (185). Using a combination of transmission electron microscopy and confocal imaging, Ulland et al. observed an increase in autophagic vesicles in microglia from Trem2^−/−^ 5xFAD mice compared to wild-type mice (137). The authors hypothesized that increased autophagic vesicles may be indicative of metabolic stress mediated, in part, by dysregulation of the mammalian target of rapamycin (mTOR) pathway, which is known for its role in controlling metabolism and autophagy (137, 186). Indeed, mTOR signaling was impaired in the microglia of Trem2^−/−^ 5xFAD mice (137). In a parallel model, using bone marrow-derived macrophages deficient of Trem2, the authors applied electrospray ionization (EIS)-MS/MS metabolomics to reveal potential mTOR-mediated energetic stress responses (137). In Trem2^−/−^ macrophages, the authors found significant decreases in uridine diphosphate-glucose, cytidine diphosphate-thanolamine, glucose-6-phosphate, fructose bisphosphate, citrate, and succinate accompanied by increases in indolacetate, glycerol-3-phosphate, malate, and fumarate when compared to wild-type cells (137). Their results suggest that TREM2 deficiency and perturbed mTOR signaling results in marked decreases in nucleotide, glycolytic, and TCA cycle metabolites with an increase in catabolic products of amino acids and phospholipids (137). The authors further investigated whether supplementation with cyclone creatine, known to increase ATP/ADP ratio (137, 187), could alleviate metabolic deficiency established in Trem2^−/−^ macrophages. Their results demonstrated that bypassing the energetic stress identified in Trem2^−/−^ macrophages by supplementation with cyclone creatine improved bioenergetics and mTOR signaling (137). These results intricately demonstrate the capability to identify biomarkers associated with AD and provide proof of concept for the ability to restore dysfunctional pathways identified using metabolomics.

## Discussion

Recent advances in the field of AD have highlighted the need to identify biomarkers for early disease diagnosis, develop new therapeutic strategies to modify or halt the disease progression, and establish models that provide reliable and strong translational value. The analysis of recent literature demonstrates that metabolomics, sparse in the past, is becoming more frequently utilized in human and animal studies extending the application to various systems including CSF, plasma, brain tissue, cells, urine, and saliva among others. Together with changes in the brain, metabolic alterations are detected in multiple tissues and fluids in AD patients suggesting a systemic nature of the disease. This underlines the potential utility of metabolomics to study pathophysiological mechanisms in living patients and establish biomarkers using readily available biofluids and tissue (e.g., plasma and skin fibroblasts).

The strength of metabolomics is in the capability of measuring the plethora of metabolites providing a snapshot of an individual’s current biological status. Since the metabolome reflects individual’s unique genomic, proteomic, and transcriptomic alterations, metabolomics can provide a global system analysis offering greater insight compared to other approaches. Application of metabolomics in animal models of AD demonstrated a significant overlap in the affected pathways identified in humans (Tables 2 and 4). This is of considerable importance providing a justification for the use of transgenic mouse models for translational research. The ability to conduct longitudinal studies assaying metabolic changes simultaneously in the brain tissue and the periphery represents an outstanding advantage of mouse models to define the relationship between preclinical biomarkers of AD vs clinical signatures. Metabolomics analysis conducted in biological samples of patients with MCI and AD identified metabolic changes associated with preclinical (18, 59, 60, 62, 132) and clinical AD (18, 58, 61, 62, 129). These findings suggest that metabolomics-based biomarkers could be used to improve disease diagnosis, which will allow to target pathways altered early in AD. As shown in mouse models of AD, monitoring therapeutic efficacy could also provide valuable insight into the mechanistic effects of potential disease-modifying drugs (134–137).

Studies using metabolomics and lipidomics have identified several metabolic pathways altered in AD including methionine, arginine, and glutamate metabolism, fatty acid biosynthesis and lipid metabolism, and mitochondrial bioenergetics (Tables 2 and 4). However, most of the published studies agreed that lipid metabolism is the most consistently altered pathway in AD pathogenesis. Toledo et al. performed one of the largest blood-based metabolomics studies in AD patients to date correlating alterations in sphingomyelins and ether-containing phosphatidylcholines with preclinical biomarker-defined AD stages and symptomatic changes with acylcarnitines, amines, and branched-chain amino acids (18). In line with these results, several other studies identified significantly altered lipids in AD (Tables 2 and 4). Specifically, changes in sphingomyelin, an important component of lipid rafts, were associated with early preclinical AD (60). Snowden et al. further demonstrated that changes in UFAs occur in the CB, which is relatively unaffected by Aβ and tau pathologies (59). These results suggested that changes in UFAs might occur early in AD development independently of Aβ and tau accumulation in the brain. Similarly, Legido-Quigley’s group observed perturbed levels of fatty acids in the plasma of AD patients including phosphatidylcholines, ChEs, and ceramides, which was further correlated with brain atrophy (63–65). Metabolomics analysis by Pan et al. of plasma and brain tissue from both humans and mice reveled that bile acids, which are important for lipid metabolism, are perturbed in AD (131). These experiments conducted using metabolomics and lipidomics clearly implicate these metabolites in the pathology of AD.

Taken together, metabolomics and lipidomics profiling has provided strong evidence that multiple factors are involved in the pathology of AD where lipid homeostasis appears to be an essential component. Lipids play a major role in normal biological functions including membrane structure, β-oxidation, cell signaling, and formation of bile acids. The first report by Alois Alzheimer described altered lipid composition in the brain tissue of AD patients (104, 105). Identification of the APOE ε4 allele as a major genetic risk factor for sporadic AD further strengthened the involvement of lipid homeostasis in AD pathogenesis. APOE is known for its primary role in lipid uptake and transport in cells. The allelic variant ε4 is associated with an increase in levels of toxic Aβ oligomers in the brain (188). In addition to APOE, lipids are also implicated in the regulation of the membrane-bound proteins associated with AD including APP, BACE1, and the presenilin proteins (189–191). Furthermore, the phosphorylation of tau protein could be influenced by various lipids (192, 193).

While metabolic profiling has provided significant insight into AD mechanisms, findings can be conflicting and inconsistent between studies. Variability in metabolic profiling arises from several factors including the experimental design, sample processing, platform selection, data analysis, and the accounting for confounders such as medication. For human studies, experimental design is often limited by the availability of samples for the analysis reducing statistical significance. Sometimes samples are not matched by age, sex, and race. Many studies lack the sufficient number of males and females, which can introduce a significant bias by overlooking sex-specific changes. Confounders, such as medications, could further complicate data analysis. However, several additional factors including diet, environment, comorbidities, and whether patients fast prior to sample collection can influence an individual’s metabolome and should also be considered. While mice allow us to control for many of these variables, several problems regarding their translational potential still exist. For instance, mice do not naturally develop AD and require the expression of human transgenes to develop symptoms. While inbreeding reduces variance in mice, this can also produce results that do not reflect a normal population. Equally important in human and mouse studies is the handling and processing of samples. Metabolic changes are dynamic and occur quickly. Therefore, careful considerations are needed to minimize metabolic alterations that could be induced during sample collection and storage. Additionally, regional tissue variation (e.g., hippocampus vs cortex) should be considered. The methodologies utilized for extraction vary depending on the nature of metabolites under investigation (e.g., lipids vs amino acids). Due to their inherent properties, different classes of metabolites require optimized extraction methods, which should be standardized in order to provide a direct comparison between results generated in different laboratories. Irrespective of the analytical platform, internal standards and reference samples are important for data normalization and quality control measurements. Finally, several multivariate statistical models exist to analyze metabolomics data (194). Proper modeling of metabolomics data is necessary to ensure accurate fitting of the data and identification of biomarkers, which is usually validated in a separate study (194). Additional post-analytical tools can be useful in the interpretation of metabolomics data for clinical application. For instance, CLIR (Collaborative Laboratory Integrated Reports; https://clir.mayo.edu/) is a web-based tool designed at the Mayo Clinic that uses multivariate pattern recognition software, which has an integrated database of clinical and laboratory data. This database is used to generate post-analytical tools capable of evaluating metabolite ratios that may discriminate between various conditions (e.g., AD vs healthy controls). The CLIR software combines multiple parameters to produce a single score that reflects the probability of having a particular condition. Using complementary analytical approaches will likely enhance the identification and accuracy of metabolic AD biomarkers and their application in the clinic.

## Conclusion

A majority of our knowledge of the molecular mechanisms of AD has derived from the identification of key genes involved in the etiology and pathology of the disease. These genes include APP, PSEN1, and PSEN2 associated with FAD. However, the majority of AD cases are sporadic with no direct genetic cause and only associated with a few identified risk factors such as the APOE ε4 allele and TREM2 variants (195). Application of current diagnostic tools strongly suggests that metabolic alterations contribute to early disease mechanisms. The identification of several metabolic networks, including lipid and amino acid metabolism, and metabolic pathways involved in glucose and energy substrate utilization provides insights into potential disease mechanism and therapeutic targets. These findings together with the identification of additional risk factors for AD, such as type 2 diabetes, strengthen the notion that AD is a metabolic disorder (196–200). Metabolomics profiling coupled to pathway analysis could aid in the understanding of the underlying disease mechanisms leading to the development of novel blood-based biomarkers for the diagnosis, prognosis, and monitoring therapeutic efficacy.

## Author Contributions

All authors listed have made a substantial, direct, and intellectual contribution to the work and approved it for publication.

## Conflict of Interest Statement

The authors declare that the research was conducted in the absence of any commercial or financial relationships that could be construed as a potential conflict of interest.
